# Use of low density lipoprotein particle number levels as an aid in statin treatment decisions for intermediate risk patients: a cost-effectiveness analysis

**DOI:** 10.1186/s12872-016-0429-6

**Published:** 2016-12-07

**Authors:** Dov Shiffman, Andre R. Arellano, Michael P. Caulfield, Judy Z. Louie, Lance A. Bare, James J. Devlin, Olle Melander

**Affiliations:** 1 Quest Diagnostics, Nichols Institute, 33608 Ortega Highway, 92675 San Juan Capistrano, CA USA; 2 Department of Clinical Sciences, Lund University, Malmö, Sweden; 3 Department of Internal Medicine, Skåne University Hospital, Malmö, Sweden

**Keywords:** Cost-effectiveness analysis, LDL-P test, Primary prevention

## Abstract

**Background:**

The 2013 ACC/AHA guideline recommended either no statin therapy or moderate-intensity statin therapy (MST) for intermediate risk patients—those with 5–7.5% 10-year risk and without cardiovascular disease (CVD), hypercholesterolemia or diabetes. The guideline further suggested that the therapy choice be based on patient-clinician discussions of risks and benefits. Since low-density lipoprotein particle (LDL-P) levels were reported to be associated with CVD independently of traditional risk factors in intermediate and low risk patients, we investigated the cost-effectiveness of using LDL-P levels to identify intermediate risk patients likely to benefit from initiating or intensifying statin therapy.

**Methods:**

We evaluated 5 care strategies for intermediate risk patients. These included the strategies suggested by the guideline: no-statin therapy and MST. We compared each of these strategies to a related strategy that incorporated LDL-P testing. No-statin therapy was compared with the strategy of MST for those with high LDL-P levels and no statin therapy for all other patients (test-and-MST). MST was compared with the strategy of high-intensity statin therapy (HST) for those with high LDL-P levels and MST for all other patients (test-and-HST). We also evaluated the strategy of HST for all. Costs (payer perspective) and utilities were assessed over a 5-year time horizon in a Markov model of 100,000 hypothetical intermediate risk patients.

**Results:**

HST dominated all other strategies, costing less and—despite causing 739 more cases of diabetes than did MST—resulting in more quality adjusted life-years (QALYs). For patient-clinician discussions that would otherwise lead to the MST strategy, we found the test-and-HST strategy reduced costs by $4.67 MM and resulted in 134 fewer CVD events and 115 additional QALYs. For patient-clinician discussions that would otherwise lead to no statin therapy, we found that the test-and-MST strategy reduced costs by $3.25 MM, resulted in 97 fewer CVD events and 44 additional QALYs.

**Conclusions:**

The HST strategy was cost saving and improved outcomes in intermediate risk patients. For patient and clinicians concerned about the adverse events associated with HST, using LDL-P levels to target intensified statin therapy could improve outcomes and reduce costs.

**Electronic supplementary material:**

The online version of this article (doi:10.1186/s12872-016-0429-6) contains supplementary material, which is available to authorized users.

## Background

Cardiovascular disease (CVD) continues to be a major cause of death in the United States. Despite an impressive ~30% decline in CVD-attributable deaths between the years 2000 and 2010, CVD still accounted for roughly 1 in 3 of all deaths in the United States in 2010 [1]. CVD prevention efforts are largely focused on improvement of modifiable risk factors such as low-density lipoprotein cholesterol (LDL-C), hypertension and smoking. The paradigm of matching the intensity of preventive efforts to the patient’s absolute risk of CVD [2, 3] allocates prevention resources to those patients who are most likely to benefit while avoiding the use of statin therapy among those who may be more likely to be harmed than to be benefitted by therapy.

The 2013 ACC/AHA guideline on the treatment of blood cholesterol to reduce atherosclerotic CVD risk in adults [4] identified 4 patient groups who would benefit from moderate- or high-intensity statin therapy: (1) patients with CVD, (2) patients with hypercholesterolemia (LDL-C >190 mg/dL), (3) patients with diabetes and (4) patients without CVD, diabetes or hypercholesterolemia but with an estimated 10-year risk of CVD ≥7.5%. There are estimated to be 12.7 million U.S. patients who would not be classified into any of these 4 groups and who have an intermediate risk of CVD (10-year risk of 5–7.5%) [4]. We focused on these intermediate risk patients because, despite the evidence showing risk reduction by statin therapy, analysis of The National Health and Nutrition Examination Survey (NHANES) indicates that most intermediate risk patients are not treated with statins [5]. And the 2013 ACC/AHA guideline suggested that for these intermediate risk patients, physicians may want to assess additional risk factors to inform treatment decisions [4].

Several studies have suggested that low-density lipoprotein particle (LDL-P) concentration is associated with CVD [6, 7]. Recently, the association between LDL-P and incident CVD was investigated among 1919 prospective, population-based cohort of patients who would not have been classified to one of the 4 statin benefit groups [8]. LDL-P was found to be associated with incident CVD events (HR = 1.40 per standard deviation) after adjusting for traditional risk factors, including standard lipids (LDL-C, high density lipoprotein cholesterol and triglycerides). After integrating the LDL-P risk [9] with traditional risk factor estimates [2] those who are believed to have a 5% 10-year risk of CVD, but are in the top decile of LDL-P, would have a 10-year CVD risk above 7.5%, a 10-year risk that could affect statin therapy decisions.

Therefore, we have modeled costs, risks and benefits in a hypothetical cohort of intermediate risk patients, and compared costs and health outcomes associated with several statin therapy strategies: no statin therapy, either moderate- or high-intensity statin therapy for all, and using the additional risk information provided by LDL-P levels as an aid in making a statin therapy decision.

## Methods

**Fig. 1:**
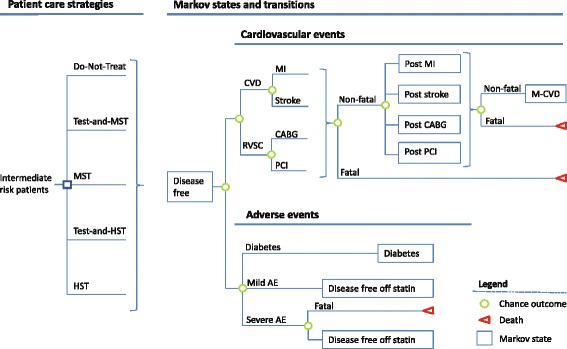
A schematic of the Markov model, indicating patient care strategies and possible transitions to cardiovascular disease and adverse event states. Five care strategies were considered: do-not-treat, no statin therapy; test-and-MST (moderate-intensity statin for those in the top decile of LDL-P levels); MST (moderate-intensity statin for all); test-and-HST (high-intensity statin for those in the top decile of LDL-P levels and moderate-intensity statin for all others); and HST (high-intensity statin for all). Abbreviations: MST, moderate-intensity statin therapy; HST, high-intensity statin therapy; MI, myocardial infarction; RVSC, revascularization, PCI, percutaneous intervention; CABG, coronary artery bypass surgery; mild AE, mild adverse events (myalgia); severe AE, (myopathy, rhabdomyolysis or hemorrhagic stroke); M-CVD, multiple CVD state. *Green* circles denote chance outcomes within a cycle; *red* triangles are terminal states. In all scenarios, patients enter the model in the disease-free state. In the first 1-year cycle individuals either remain in the disease-free state or experience a clinical event (MI, stroke, CABG, PCI, mild or severe AE, diabetes or death)

We developed a cohort-level Markov state-transition model to evaluate the cost effectiveness of patient-care strategies for primary prevention of CVD in intermediate risk patients (Fig. 1). The model calculated the costs, benefits and harms for a hypothetical cohort of 100,000 patients at intermediate risk for CVD [4]: men and women aged 40–75 years with LDL-C 70–189 mg/dL, an estimated 10-year CVD risk between 5% and 7.5%, and without clinical CVD or diabetes.

We used the model to evaluate the costs and utilities of 5 patient-care strategies. Two of the strategies we considered were a strategy without statin therapy (do-not-treat) and one in which all patients receive moderate-intensity statin therapy (MST), both of which the 2013 ACC/AHA guideline suggested as reasonable choices for intermediate risk patient. Additionally, we considered 2 strategies that incorporated LDL-P testing: (1) treat those in the top decile of LDL-P levels with MST (test-and-MST) and (2) treat those in the top decile of LDL-P levels with HST and all others with MST (test-and-HST). Although the 2013 ACC/AHA guideline notes that potential adverse effects from high-intensity statin therapy (HST) may outweigh the potential for cardiovascular risk-reduction benefit in intermediate risk patients, we also included a strategy that treated all intermediate risk patients with HST in order to cover the full range of statin therapy intensity in the strategies considered. For each strategy, we calculated the costs and outcomes from a payer’s perspective using 1-year cycles over a 5-year time horizon—a time horizon that would be of interest to third-party payers due to high turnover rates in their insured populations. Outcomes included CVD events (nonfatal and fatal myocardial infarction [MI] or stroke), revascularization events (coronary artery bypass surgery [CABG] or percutaneous intervention [PCI]), statin-related adverse events (diabetes, severe adverse events or mild adverse events), and the number of quality-adjusted life years (QALYs).

We modeled a population that, based on traditional risk factors, had a 10-year CVD risk estimate with a flat distribution and a range of 5–7.5% (average risk of 6.25%). Elevated LDL-P levels were found to be associated with increased risk of CVD (HR = 1.40 per standard deviation) in a population with intermediate or low risk after adjustment for established risk factors, including standard lipids [8]. We assumed that the LDL-P distribution in our study population was similar to the distribution found in Melander et al. [8], that is, in men and women with LDL-C level of 70–189 mg/dL, an estimated 10-year CVD risk lower than 7.5%, and without clinical CVD or diabetes. We converted the reported LDL-P risk per one standard deviation increase to risk per one decile of LDL-P level based on the assumption that the risk associated with LDL-P is log-linear over the entire LDL-P range. Then we used the method of Kooter et al. [9] to modify the 10-year risk estimates based on LDL-P levels. The average redefined 10-year CVD risk was 10.52% for those in the top LDL-P decile, 3.23% for those in the bottom decile and 6.09% for those in deciles 2 through 9. After redefining risk estimates based on LDL-P levels, all patients in the top decile of LDL-P—even those considered to be at 5% 10-year risk of CVD based on traditional risk factors—would be above the 7.5% 10-year risk threshold recommended for statin therapy.

The annual event rates—transition probabilities between states—are based on published literature (Table 1). When necessary, cumulative event rates were used to estimate annual event rates by assuming that the times to event had exponential distribution with constant hazard. Specifically, hazards were calculated from the cumulative event rates and follow-up years based on the survival equation for exponential distribution with constant hazard. Then, the estimated hazards were used to calculate the annual event rates [10]. The hazard for the combined CVD and revascularization events was the sum of the CVD hazard and the product of CVD hazard and the ratio of revascularization to CVD events in the placebo arm of the JUPITER trial [11]. We estimated the hazards for CVD post CABG or CVD post PCI as the difference between hazards for CVD and mortality after CABG or PCI in the Syntax trial [12]. Patients who had a CVD or a revascularization event received statin therapy in our model and the risk of recurrent events was based on the risk observed in the treatment arms of secondary prevention studies [12–15]. We assumed that a third CVD event in any patient would be fatal. The model includes three categories of adverse events due to statin therapy: mild (myalgia), severe (rhabdomyolysis) and statin-induced diabetes. Individuals with mild or severe adverse events were assumed to discontinue statin therapy. Patients who developed diabetes were assumed to be treated with high-intensity statin therapy [4]. The model does not assume increased risk of CVD or revascularization as a result of new-onset diabetes, since the effect of new-onset diabetes on the risk of CVD events has been reported to be negligible over a 5-year time-horizon [16–19]. To account for adherence to statin therapy, we assumed that non-adherence is the sum of 2 non-adherent groups: patients with mild adverse events (who were assumed to discontinue statin therapy in the first year) and patients who discontinued statin therapy within the first year for unspecified reasons. Non-adherent patients were assumed to proceed through the remaining four years off statin therapy. We assumed non-adherence was equal in all care strategies.

**Table 1:** Event rates and transition probabilities

Event type	Cumulative rate (years)	Transition probability (range)	Distribution	References
CVD	0.0625 (10)	0.00643	No change	By design
Revascularization	0.0625 (10)	0.00643	No change	Derived from CVD (above) and MI, stroke and revascularization rate in JUPITER, Ridker et al. NEJM 2008 [11]
MI		0.0037 (±20%)	β	Ridker et al. NEJM 2008 [11]
Stroke		0.0034 (±20%)	β	Ridker et al. NEJM 2008 [11]
Revascularization		0.0071 (±20%)	β	Ridker et al. NEJM 2008 [11]
Recurrent MI	0.144 (7)	0.022 (±20%)	β	Cannon et al. NEJM 2015 [13]
MI post stroke		0.0074 (±20%)	β	Greisenegger et al. Stroke 2015 [14]
Recurrent stroke		0.023 (±20%)	β	Greisenegger et al. Stroke 2015 [14]
CVD post CABG	0.269 CVD (5) 0.114 mortality (5)	0.0377 (±20%)	β	Mohr et al. Lancet 2013 [12]
CVD post PCI	0.373 CVD (5) 0.139 mortality (5)	0.0615 (±20%)	β	Mohr et al. Lancet 2013 [12]
Death post MI	0.222 (7)	0.0352 (±20%)	β	Cannon et al. NEJM 2015 [13]
Death post-stroke		0.0649 (±20%)	β	Greisenegger et al. Stroke 2015 [14]
Death post CABG	0.114 (5)	0.0239 (±20%)	β	Mohr et al. Lancet 2013 [12]
Death post PCI	0.139 (5)	0.0295 (±20%)	β	Mohr et al. Lancet 2013 [12]
Death post multiple CVD		0.1 (±20%)	β	Law et al. Arch Int Med 2002 [15]
Death post severe adverse event		0.09 (±20%)	β	Lee et al. Circulation 2010 [32]
Diabetes from high-intensity statin		0.003 (±20%)	β	Stone et al. Circulation 2014 [4]
Diabetes from moderate-intensity statin		0.001 (±20%)	β	Stone et al. Circulation 2014 [4]
Mild adverse events from statin		0.056 (0.0001–0.175)	β	Kashani et al. Circulation 2006 [41]
Severe adverse events from statin		0.0001 (±20%)	β	Stone et al. Circulation 2014 [4]

When published event rate were reported by others as cumulative rates, the cumulative rate is shown in the table, and converted to 1-year event rates by assuming constant hazard and exponential distribution of time to event (see Methods)

Costs from published sources (Table 2) were inflated to 2014 levels using the seasonally adjusted medical care component of the consumer price index to the year 2014 [20]. We assumed that the utility of being in a disease-free state while not taking a statin was 1 (perfect health) in this primary prevention population [21]. The utility of death was set to zero. We also included a disutility for taking a statin pill every day [21]. Health utilities for MI, stroke, angina and diabetes were based on published values [22]. Future costs and utilities were discounted at an annual rate of 3% as recommended by the US Panel on Cost-Effectiveness in Health and Medicine [23]. The Consolidated Health Economic Evaluation Reporting Standards (CHEERS) were followed in reporting this economic evaluation [24].

**Table 2:** Model Parameters

Parameter	Base-Case (range)	Distribution	References
LDL-P relative risk (per SD)	1.40 (1.12–1.75)	Log normal	Melander et al. JACC 2015 [8]
Fraction of CABG in revascularization	0.2 (±20%)	β	Ohsfeldt et al. J Med Econ 2010 [35]
Fraction of fatal MI among MI	0.125 (±20%)	β	Choudhry et al. JACC 2011 [36]
Fraction of fatal stroke among stroke	0.132 (±20%)	β	Choudhry et al. JACC 2011 [36]
Fraction discontinuing statin therapy^a^	0.254 (0–0.444)	β	Pletcher et al. CircCQO 2014 [31]
Effect of Interventions			
High-intensity statin			
MI	0.46 (0.30–0.70)	Log normal	Choudhry et al. JACC 2011 [36], Ridker et al. NEJM 2008 [11]
Revascularization	0.54 (0.41–0.72)	Log normal	Choudhry et al. JACC 2011 [36], Ridker et al. NEJM 2008 [11]
Stroke	0.52 (0.34–0.79)	Log normal	Choudhry et al. JACC 2011 [36], Ridker et al. NEJM 2008 [11]
Moderate-intensity statin			
Coronary Artery Disease	0.75 (0.71–0.78)	Log normal	Pandya et al. JAMA 2015 [21]
Stroke	0.83 (0.76–0.87)	Log normal	Pandya et al. JAMA 2015 [21]
State utilities			
Disease free off statins	1	unchanged	Assumption
Disease free taking statins	0.998 (0.991–1.0)	β	Pandya et al. JAMA 2015 [21]
Post MI	0.778 (0.575–0.843)	β	Sullivan et al. Med Decis Making 2006 [22]
Post Stroke	0.768 (0.463–0.816)	β	Sullivan et al. Med Decis Making 2006 [22]
Post PCI or CABG	0.768 (0.517–0.827)	β	Sullivan et al. Med Decis Making 2006 [22]
Multiple CVD^b^	0.605 (±20%)	β	Calculated from Sullivan et al. Med Decis Making 2006 [22]
Diabetes	0.800 (0.708–0.844)	β	Sullivan et al. Med Decis Making 2006 [22]
Mild adverse events (disutility)	0.005 (±20%)	β	Lee et al. Circulation 2010 [32]
Severe adverse events (disutility)	0.038 (±20%)	β	Lee et al. Circulation 2010 [32]
Costs (2014 US dollars)			
LDL-P test	42.29 (±20%)	γ	CMS fee schedule [37]
Nonfatal MI (1^st^ year)	69,819 (±20%)	γ	O’Sullivan et al. Pharmacoeconomics 2011 [38]
Fatal MI	19,373 (±20%)	γ	O’Sullivan et al. Pharmacoeconomics 2011 [38]
Nonfatal stroke (1^st^ year)	23,021 (±20%)	γ	O’Sullivan et al. Pharmacoeconomics 2011 [38]
Fatal stroke	11,951 (±20%)	γ	O’Sullivan et al. Pharmacoeconomics 2011 [38]
CABG (1^st^ year)	41,388 (±20%)	γ	O’Sullivan et al. Pharmacoeconomics 2011 [38]
PCI (1^st^ year)	38,998 (±20%)	γ	O’Sullivan et al. Pharmacoeconomics 2011 [38]
Diabetes (diagnosis)	138.18 (±20%)	γ	Choudhry et al. JACC 2011 [36]
Severe adverse events	7,852 (±20%)	γ	Lee et al. Circulation 2010 [32]
Mild adverse events	199.32 (±20%)	γ	Lee et al. Circulation 2010 [32]
Low/Moderate-intensity statin therapy (annual)	48.00 (±20%)	γ	www.healthwarehouse.com [39]
High-intensity statin therapy (annual)	91.00 (±20%)		www.healthwarehouse.com [39]
MI (subsequent years, annual)	507.83 (±20%)	γ	Choudhry et al. JACC 2011 [36]
CABG or PCI (subsequent years, annual)	507.83 (±20%)	γ	Assumed to be equal to MI
Stroke (subsequent years, annual)	20263.60 (±20%)	γ	Choudhry et al. JACC 2011 [36]
Multiple CVD state (subsequent years, annual)	9968.34 (±20%)	γ	O’Sullivan et al. Pharmacoeconomics 2011 [38]
Diabetes (annual)	2660.67 (±20%)	γ	Soni, AHRQ statistical brief #304. 2010 [40]

^a^Statin discontinuation includes discontinuation due to adverse events

^b^Multiple CVD utility is assumed to be the utility of post-MI state squared

TreeAge Pro software 2015 (TreeAge Software, Williamstown, MA) was used for modeling. Costs and utilities for the base-case strategies were calculated using a half-year correction. The effect of varying input parameters was explored by deterministic sensitivity analyses in which input parameters were individually changed to the upper and lower values of their range. The ranges of input parameters were based on published literature where available, or were set to ±20% of base values for parameters without published ranges (Table 2). Monte Carlo simulations were performed to conduct probabilistic sensitivity analyses where input parameters were simultaneously varied by sampling values from the probability distribution of each parameter. Beta distributions were used for transition probabilities and for utilities. Gamma distributions were used for costs. Log-normal distributions were used for hazard ratios. The sampling process was repeated 10,000 times.

## Results

We estimated the costs and utilities for 5 care strategies (Fig. 1) in a hypothetical cohort of 100,000 individuals at intermediate risk (5–7.5% 10-year risk) of CVD over a 5-year time horizon. When information from LDL-P levels was used to modify CVD risk estimates based traditional risk factors, the average 10-year risk estimate of the cohort remained unchanged. We validated this aspect of our model by changing LDL-P hazard ratio parameter in the model from 1.0 to 2.0 per standard deviation without observing any change in the number of patients experiencing a first CVD event or revascularization event over 10 years.

The most intensive strategy we considered—primary prevention with HST for all patients in this cohort—dominated all other strategies. It resulted in 784 fewer CVD events, and 475 fewer revascularization events than the MST strategy, and HST had the lowest cost, $258.46 million (Additional file 1: Tables S1 and S3). However, the HST strategy also caused more diabetes than did any of the other strategies, resulting in 739 additional diabetes diagnoses compared with MST for a net gain of 512 QALYs.

For those patients and clinicians whose discussions lead to a no-statin-therapy decision, the alternative strategy evaluated was to treat only those in the top decile of LDL-P levels with MST (test-and-MST). When comparing these 2 strategies the test-and-MST strategy dominated the do-not-treat strategy (Tables 3 and 4). The test-and-MST strategy reduced costs by 3.25 million dollars and resulted in 97 fewer CVD and 97 fewer revascularization events. Although statin therapy in the test-and-MST strategy resulted in statin-induced diabetes in 36 patients and 4 severe adverse events (rhabdomyolysis) compared with the do-not-treat strategy, the test-and-MST strategy resulted in better outcomes, adding 44 QALYs compared with the do-not-treat strategy.

**Table 3:** Base case results for 5 statin treatment strategies in 100,000 hypothetical intermediate risk patients

Strategy	CVD (events, *n*)	RVSC (events, *n*)	Mild Adverse Events (*n*)	Severe Adverse Events (*n*)	Diabetes diagnoses (*n*)	Cost ($1000)	QALYs	Cost/QALY ($)	ΔCost ($1000)	ΔQALY	ICER
HST	2,527	2,252	5,600	37	1,107	258,460	460,516	561	−44,755^a^	512^a^	Dominant^b^
Test-and-HST	3,177	2,646	5,600	37	442	298,547	460,119	649	−4,668^a^	115^a^	Dominant
MST	3,311	2,727	5,600	37	368	303,215	460,004	659	Reference	Reference	
Test-and-MST	3,787	3,197	560	4	36	336,633	460,162	732	−3,246^c^	44^c^	Dominant^d^
Do-not-treat	3,884	3,294	0	0	0	339,879	460,118	739	Reference	Reference	

Care strategies: do-not-treat, no statin therapy; test-and-MST, moderate-intensity statin therapy for those in the top decile of LDL-P levels; MST, moderate-intensity statin therapy for all; test-and-HST, high-intensity statin therapy those in the top decile of LDL-P levels and moderate-intensity statin therapy for all other; HST, high-intensity statin therapy for all

*CVD*, cardiovascular disease, *RVSC*, revascularization

^a^Compared with MST

^b^Dominates both MST and do-not-treat

^c^Compared with do not treat

^d^ICER equals 211,456 ($/QALY) when compared with MST

**Table 4:** Base-case results: Pairwise comparison

	Test-and-MST	Do-not-treat	Increment	Test-and-HST	MST	Increment
CVD events (*n*)	3787	3884	(97)	3177	3311	(134)
RVSC events (*n*)	3197	3294	(97)	2646	2727	(81)
Mild Adverse Events (*n*)	560	0	560	5600	5600	0
Severe Adverse Events (*n*)	4	0	4	37	37	0
Diabetes diagnoses (*n*)	36	0	36	442	368	74
Cost ($, Millions)	336.63	339.88	(3.25)	298.55	303.22	(4.67)
QALYs	460,162	460,118	44	460,119	460,004	115

Care strategies: do-not-treat, no statin therapy; test-and-MST, moderate-intensity statin therapy for those in the top decile of LDL-P levels; MST, moderate-intensity statin therapy for all; test-and-HST, high-intensity statin therapy those in the top decile of LDL-P levels and moderate-intensity statin therapy for all other; HST, high-intensity statin therapy for all

*CVD* cardiovascular disease, *RVSC* revascularization

For those patients and clinicians whose discussions lead to an MST decision, the alternative strategy evaluated was to treat those in the top decile of LDL-P levels with HST and treat the rest with MST (test-and-HST). The test-and-HST strategy reduced costs by 4.67 million dollars and resulted in 134 fewer CVD and 81 fewer revascularization events when compared to the MST strategy. Despite the occurrence of 74 additional cases of statin-induced diabetes diagnosis, the test-and-HST strategy improved overall outcomes by adding 115 QALYs compared with the MST strategy.

**Fig. 2:**
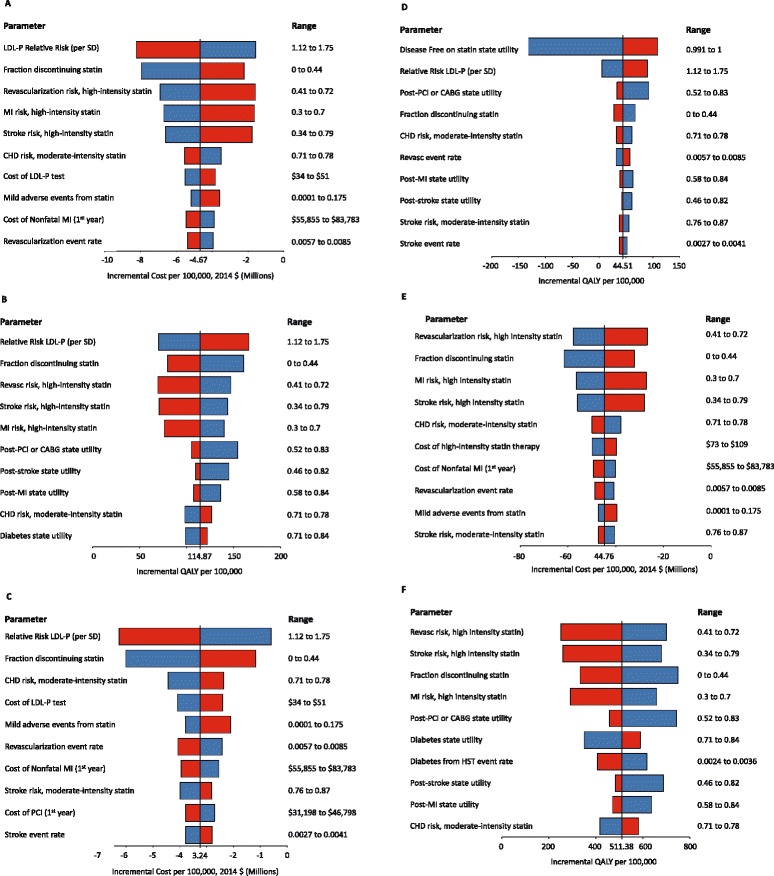
Deterministic sensitivity analysis of patient-care strategies. The incremental costs and incremental QALYs were assessed for a cohort of 100,000 patients using the upper range (*red* bar) and lower range (*blue* bar) of each parameter while keeping all other parameters at their base-case value. The results for the 10 parameters that caused the largest changes are reported. Panel **a** Incremental costs for test-and-HST vs. MST; Panel **b** Incremental QALYs for test-and-HST vs. MST; Panel **c** Incremental costs for test-and-MST vs. do-not-treat; Panel **d** Incremental QALYs for test-and-MST vs. do-not-treat; Panel **e** Incremental costs for HST vs. MST; Panel **f** Incremental QALYs for HST vs. MST

We investigated the robustness of the model outcomes in a deterministic sensitivity analysis that changed each input parameter to its highest and its lowest possible values (Tables 1 and 2) while keeping all other input parameters at their base-case value (Fig. 2, and Additional file 1: Tables S2, S3 and S4). We found that the cost-savings and the increase in QALYs were maintained for all parameters except for the utility of being disease free while taking a statin pill daily. At the lowest end of the range for this parameter (0.99), the test-and-MST strategy resulted in fewer QALYs than did the do-not-treat strategy (Fig. 2 Panel d).

**Fig. 3:**
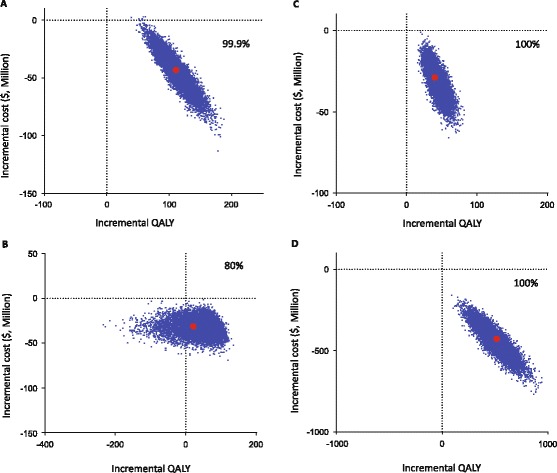
Probabilistic sensitivity analyses of patient-care strategies for a cohort of 100,000 patients. In this Monte Carlo simulation all parameters are simultaneously varied from their base-case values by sampling from probability distributions (Tables 1 and 2). The sampling process was repeated 10,000 times. The percent of the samplings that resulted in a test strategy with more QALYs at a lower cost compared with the comparable no-test strategy is shown in each panel. Each *blue* dot represents the result of one sampling of the parameters. The red dot represents the result using base-case parameters values. To clearly visualize the distributions of the simulation results, a randomly selected 1,000 (of the 10,000) samplings are plotted as blue dots in each panel. Panel **a** test-and-HST vs. MST; Panel **b** test-and-MST vs. Do-not-treat; Panel **c** test-and-MST vs. Do-not-treat, with the utility of being disease-free while taking a statin pill daily fixed at the base-case value. Panel **d** HST vs. MST

We also investigated the sensitivity of the model outcomes to the input parameters by varying the parameters in a Monte-Carlo simulation. That is, we simultaneously varied all input parameters by sampling their values from probability distributions chosen to reflect the uncertainty in the parameter estimates (Table 1 and 2). This sampling process was repeated 10,000 times. In this probabilistic sensitivity analysis, the test-and-HST strategy dominated the MST strategy for all 10,000 iterations of the simulations (Fig. 3, Panel a). A similar analysis of the test-and-MST strategy and the do-not-treat strategy revealed that the MST strategy dominated the do-not-treat strategy in 80% of the iterations (Fig. 3 Panel b). For the remaining 20% of iterations, the test-and-MST strategy resulted in fewer QALYs compared with the do-not-treat strategy. Since the deterministic sensitivity analysis indicated that the reduced QALYs were due to the slight decrease in utility assigned to taking a statin pill daily, we conducted an exploratory analysis, and found that when the probabilistic sensitivity analysis used only the base-case value (0.998) for the utility of being disease-free while taking statin pill daily, the test-and-MST strategy dominated the do-not-treat strategy in all of the simulations (Fig. 3, Panel c).

## Discussion

We assessed the cost-effectiveness of 5 care strategies for patients at intermediate risk of CVD and found that treating all such patients with HST resulted in lower costs and fewer CVD or revascularization events than did any of the other strategies. Therefore, although the ACC/AHA guideline [4] suggested either MST or no statin therapy as potential care strategies for primary prevention in intermediate risk patients, our analysis indicates that patients and clinicians might also want to consider an HST strategy in their discussion. A recent study [25] found that HST for all men aged 45–75 and women aged 55–75 regardless of their risk level dominated other risk-based strategies over a 30-year time-horizon. Our findings are consistent with this study, while also limiting the analysis to a 5-year time-horizon, a time-horizon relevant to insurance providers, and focusing on intermediate risk patients for whom the ACC/AHA guideline provides room for patient-physician discussions in making treatment decisions.

Since the ACC/AHA guideline suggested that information from additional risk assessment could help inform statin initiation decisions for intermediate risk patients, we considered strategies that identified patients with elevated (top decile) LDL-P levels. Although patient-physician discussions may involve a rational comparison of costs and benefits, the actual weights that a particular patient assigns to the benefits, risks and costs are very much a matter of personal preference and, as such, cannot be determined analytically. However, when these discussions would benefit from additional information about a patient’s risk, an analytical approach can be used to assess whether providing that information is likely to be cost effective, which has been the motivation for our analysis. We found that for patients and clinicians, whose discussions would otherwise lead to either a do-not-treat or an MST decision, adopting a strategy that includes a more intensive statin therapy for patients in the top LDL-P decile could be less costly, result in more QALYs and lead to fewer CVD or revascularization events over a 5-year horizon.

For intermediate risk patients and their physicians who would otherwise choose a do-not-treat or MST strategy, a decision to choose a more aggressive strategy would probably require compelling additional information about the patient’s risk level. Therefore, in the test and treat strategies, only those in the top decile of LDL-P would be treated. For this LDL-P level, even those with the lowest risk in the intermediate risk group (5% 10-year risk) would have a 10-year risk >7.5%—a 10-year risk that would put them above the risk threshold at which the ACC/AHA guideline suggested considering high-intensity statin therapy. After incorporating the LDL-P level into the risk prediction model, while some of the patients have greater than 7.5% risk based on the new information, some have less than 5% risk; our model incorporated this lower risk estimate into the costs and utilities calculations. However, the model did not suggest a change in the care strategy for these lower risk patients.

Several studies have suggested that statins can be cost-effective at lower risk thresholds lower than those in the ACC/AHA 2013 guideline (see Deano et al. [26] for a review). An early cost-effectiveness study by Pignone et al. [27] investigated the addition of statin therapy to aspirin therapy in primary prevention. Although annual statin costs at the time of that study were about 10-fold greater than current costs, they found that if patients with 7.5% 10-year risk or greater were treated, the addition of statin therapy would cost $56,200 per QALY gained over a life-time horizon. A more recent study suggested that if the ATP III guidelines were modified to suggest statin therapy for all primary prevention patients with >7.5% 10-year risk and LDL-C >130 mg/dL, the cost would be $50,000 per QALY gained over a 30-year time horizon [28]. A follow-up analysis using the same model [29] analyzed the effect of the reduced cost of generic statins on the cost per QALY gained even if patients with only modest LDL-C elevations were treated. Recently, Pandya et al. [21] investigated the 2013 ACC/AHA guideline thresholds and reported that treating patients with a 3% or greater 10-year risk of CVD would cost $150,000 per QALY gained.

This current study differs from the Pandya et al. study [21] in both time-horizon and its treatment of risk thresholds. We considered a 5-year time horizon for the analysis, rather than a lifetime horizon, to better align with the payer perspective of the analysis (private payers typically do not have lifetime responsibility for a patient). Had this analysis been conducted from a societal perspective and lifetime time-horizon, we believe that the strategies that incorporate LDL-P testing would have had even more favorable costs and outcomes because (1) more events would be prevented over a lifetime in patients who were tested and consequently treated with statins and (2) a societal perspective analysis could include the cost of lost productivity associated with cardiovascular events. However, a lifetime perspective would result in more diagnoses of diabetes and diabetes complications among those treated with HST, which could make LDL-P testing somewhat less favorable for patients in the test and HST strategy. Nevertheless, the strategies that include HST are likely to retain their advantage since HST for all dominated risk based strategies over a 30 year time-horizon for all for all men aged 45–75 and women aged 55–75 [25]. Rather than considering whether the guideline treatment thresholds are appropriate, we focused on patients at intermediate risk and the treatment strategies suggested by the 2013 ACC/AHA guideline for patients with a 5–7.5% 10-year risk for CVD events, and how additional information about risk of CVD events bases on an independent risk factor might modify the choice of treatment strategies.

To account for uncertainty in the estimates of base-case parameters, we investigated the effects of changing parameter values using probabilistic sensitivity analyses. One interesting result of these analyses was that although the test-and-MST strategy dominated the do-not-treat strategy most of the time, the test-and-MST strategy resulted with fewer QALYs than the do-not-test strategy in about 20% of the simulations. A deterministic sensitivity analysis indicated that for these 20% of the simulations, the utility value of being disease free while taking a statin pill daily was probably drawn from the low end of the probability distribution. Although the magnitude of the disutility for taking a statin was modest, the effect of this small disutility was amplified because ~10% of the patients in the test-and-MST strategy were taking a statin pill daily whereas almost no statins were used in the do-not-treat strategy other than patients who had a CVD or a revascularization event. The disutility of taking statins had no noticeable effect when we compared the MST strategy with the test-and-HST strategy because all patients used statins in both strategies. The effect of the disutility of a daily statin pill on reducing the cost-effectiveness of statin treatment in the primary prevention of CVD is consistent with other studies of the cost-effectiveness of statin in the primary prevention of CVD [21, 30–32]. These studies found that a higher CVD risk threshold for statin treatment was needed to offset a large statin use disutility.

Because pill taking disutility can differ substantially from person to person, clinicians and patients should consider individual preferences selecting a care strategy. Pill taking disutility is based on questionnaires that assess personal preferences, and an accurate assessment of a small disutility may be influenced by the numeracy of the surveyed individuals [33]. Although some individuals may be willing to trade off a small risk of death for avoiding taking a pill daily, in a recent study [33] >60% of individuals were unwilling to accept any risk of death for not taking a daily pill—that is, their pill taking disutility was 0. These individuals might be the most appropriate group for test-and-treat strategies since they would be most likely to accept and then adhere to statin therapy.

One limitation of this study is that the model included several simplifying assumptions. For example, the outcome of a third CVD event was assumed to be fatal, all patients with newly diagnosed diabetes were assumed be treated with statins, and all statin-related adverse events were assumed to occur within the first year of taking statins. In general, these simplifications eliminated rare states that, because of their rarity, would have a small effect on the model outcome and have little published support for parameter estimates. We validated the internal consistency of the model by exploring the outcomes of extreme parameter values. However, we were unable to perform external validation by comparing the model outcomes to a real-life study because no outcome study of statin use in patients with estimated 5–7.5% 10-year risk of CVD has been published. Moreover, this study is limited to a primary prevention population with intermediate risk of CVD. Thus, we did not consider patient treatment strategies that could involve angiography-based plaque imaging risk assessment [34]. Another limitation is that, although we tried to limit the number of studies used as a source of model parameters, we were unable to determine all model parameters from a single study. Base-case parameter variability could change the model outcome, in particular, if the effect size of the association between LDL-P level and CVD events was overestimated, then those with high LDL-P levels would have fewer CVD events and the number of events prevented by treating this high LDL-P group would be smaller. We assumed that the LDL-P distribution in our study (individuals with an estimated 5–7.5% 10-year risk) is similar to the distribution in those with <7.5% 10-year risk in whom the LDL-P risk estimate were assessed. Although these populations are similar, changes in LDL-P distribution or LDL-P risk assessment could change the model outcomes.

## Conclusions

In conclusion, this study indicates that HST is the preferred care strategy in intermediate risk patients. For those patients and physicians who decide to avoid HST, LDL-P testing can assist in making statin treatment decisions and is likely to reduce CVD events and to be cost saving.

## Additional file

Additional file 1: Table S1. Health outcomes and costs for different statin treatment strategies for primary prevention of cardiovascular disease in 100,000 hypothetical intermediate risk patients: Base-case analysis. **Table S2.** Deterministic Sensitivity Analysis. Incremental Cost and Incremental QALY per 100,000 patients for all parameters for the Test and HST strategy 1 compared with the MST strategy. **Table S3.** Deterministic Sensitivity Analysis. Incremental Cost and Incremental QALY per 100,000 patients for all parameters for the Test and MST strategy 1 compared with the Do-Not-Treat strategy. **Table S4.** Deterministic Sensitivity Analysis Incremental Cost and Incremental QALY per 100,000 patients for all parameters for the HST strategy compared 1 with the MST strategy. (DOCX 67 kb)

## Abbreviations

CVD: Cardiovascular disease
HST: High-intensity statin
LDL-P: Low density lipoprotein particle
MST: Moderate-intensity statin
QALY: Quality adjusted life-year
