# Supplementary material for: Use of low density lipoprotein particle number levels as an aid in statin treatment decisions for intermediate risk patients: a cost-effectiveness analysis
Source: BMC Cardiovasc Disord. 2016 Dec 7;16:251. doi: 10.1186/s12872-016-0429-6 (PMC5142314; doi:10.1186/s12872-016-0429-6)
Supplement: Additional file 1: Table S1. — Health outcomes and costs for different statin treatment strategies for primary prevention of cardiovascular disease in 100,000 hypothetical intermediate risk patients: Base-case analysis. Table S2. Deterministic Sensitivity Analysis. Incremental Cost and Incremental QALY per 100,000 patients for all parameters for the Test and HST strategy 1 compared with the MST strategy. Table S3. Deterministic Sensitivity Analysis. Incremental Cost and Incremental QALY per 100,000 patients for all parameters for the Test and MST strategy 1 compared with the Do-Not-Treat strategy. Table S4. Deterministic Sensitivity Analysis Incremental Cost and Incremental QALY per 100,000 patients for all parameters for the HST strategy compared 1 with the MST strategy. (DOCX 67 kb) [file 12872_2016_429_MOESM1_ESM.docx]

| **Strategy** | **CVD**  **events** | **RVSC**  **events** | **Mild**  **adverse**  **events** | **Severe**  **adverse**  **events** | **Diabetes**  **diagnoses** | **Cost**  **(MM USD)** | **QALYs** | **$/**  **QALY** | **ICER** |
| --- | --- | --- | --- | --- | --- | --- | --- | --- | --- |
| High-intensity statin, no LDL-P test (HST) | 2527 | 2252 | 5600 | 37 | 1,107 | 258.46 | 460,516 | 561 | **Dominant** |
| Test and treat only LDL-P top decile with moderate-intensity statin (Test-and-MST) | 3787 | 3197 | 560 | 4 | 36 | 336.63 | 460,162 | 732 | Dominated |
| Test and treat LDL-P top decile with high-intensity statin, moderate-intensity statin for all others (Test-and-HST) | 3177 | 2646 | 5600 | 37 | 442 | 298.55 | 460,119 | 649 | Dominated |
| No statin, No LDL-P test (Do-not-treat) | 3884 | 3294 | 0 | 0 | 0 | 339.88 | 460,118 | 739 | Dominated |
| Moderate-intensity statin, no LDL-P test (MST) | 3311 | 2727 | 5600 | 37 | 368 | 303.22 | 460,004 | 659 | Dominated |

**Table S1. Health outcomes and costs for different statin treatment strategies for primary prevention of cardiovascular disease in 100,000 hypothetical intermediate-risk patients: Base-case analysis**

CVD, cardiovascular disease; RVSC, revascularization.

**Table S2. Deterministic Sensitivity Analysis:** Incremental Cost and Incremental QALY per 100,000 patients for all parameters for the Test and HST strategy compared with the MST strategy.

|  |  | **Low range** | | |  | **High range** | | |
| --- | --- | --- | --- | --- | --- | --- | --- | --- |
| **Parameter** | **Base**  **Value** | **Value** | **Inc. Cost**  **($, MM)** | **Inc.**  **Utility** |  | **Value** | **Inc. Cost**  **($, MM)** | **Inc.**  **Utility** |
| **Effect of interventions** |  |  |  |  |  |  |  |  |
| High-intensity statin (risk reduction) |  |  |  |  |  |  |  |  |
| MI | 0.46 | 0.30 | -6.70 | 140 |  | 0.70 | -1.64 | 76 |
| Revascularization | 0.54 | 0.41 | -6.91 | 147 |  | 0.72 | -1.58 | 69 |
| Stroke | 0.52 | 0.34 | -6.61 | 144 |  | 0.79 | -1.77 | 71 |
| Moderate-intensity statin (risk reduction) |  |  |  |  |  |  |  |  |
| CHD | 0.75 | 0.71 | -3.49 | 98 |  | 0.78 | -5.55 | 127 |
| Stroke | 0.83 | 0.76 | -3.93 | 103 |  | 0.87 | -5.09 | 121 |
| **Ratios** |  |  |  |  |  |  |  |  |
| Relative Risk LDL-P (per SD) | 1.4 | 1.12 | -1.58 | 70 |  | 1.75 | -8.24 | 166 |
| Fraction of CABG in revascularization | 0.2 | 0.16 | -4.67 | 114 |  | 0.24 | -4.67 | 114 |
| Fraction of fatal MI among MI | 0.125 | 0.10 | -4.74 | 112 |  | 0.15 | -4.59 | 117 |
| Fraction of fatal stroke among stroke | 0.132 | 0.11 | -4.75 | 112 |  | 0.16 | -4.59 | 117 |
| Fraction discontinuing statin therapy | 0.31 | 0 | -7.96 | 161 |  | 0.44 | -2.21 | 80 |
| **State utilities** |  |  |  |  |  |  |  |  |
| Disease free taking statins | 0.998 | 0.991 | -4.67 | 113 |  | 1.0 | -4.67 | 115 |
| Post-MI | 0.778 | 0.58 | -4.67 | 136 |  | 0.84 | -4.67 | 107 |
| Post-Stroke | 0.768 | 0.46 | -4.67 | 145 |  | 0.82 | -4.67 | 110 |
| Post-PCI or CABG | 0.768 | 0.52 | -4.67 | 154 |  | 0.83 | -4.67 | 105 |
| Multiple CVD^†^ | 0.605 | 0.48 | -4.67 | 116 |  | 0.73 | -4.67 | 112 |
| Diabetes | 0.8 | 0.71 | -4.67 | 99 |  | 0.84 | -4.67 | 122 |
| Mild adverse events (disutility) | 0.005 | -0.006 | -4.67 | 114 |  | -0.004 | -4.67 | 114 |
| Severe adverse events (disutility) | 0.038 | -0.046 | -4.67 | 114 |  | -0.030 | -4.67 | 114 |
| **Costs (2014 USD)** |  |  |  |  |  |  |  |  |
| LDL-P test | 42 | 34 | -5.51 | 114 |  | 51 | -3.82 | 114 |
| Nonfatal MI (1^st^ year) | 69,819 | 55,855 | -3.88 | 114 |  | 83,783 | -5.46 | 114 |
| Fatal MI | 19,373 | 15,499 | -4.63 | 114 |  | 23,248 | -4.71 | 114 |
| Nonfatal stroke (1^st^ year) | 23,021 | 18,417 | -4.45 | 114 |  | 27,626 | -4.88 | 114 |
| Fatal stroke | 11,951 | 9,561 | -4.64 | 114 |  | 14,341 | -4.70 | 114 |
| CABG (1^st^ year) | 41,388 | 33,111 | -4.55 | 114 |  | 49,666 | -4.79 | 114 |
| PCI (1^st^ year) | 38,998 | 31,198 | -4.21 | 114 |  | 46,798 | -5.12 | 114 |
| Diabetes (diagnosis) | 138 | 111 | -4.67 | 114 |  | 166 | -4.67 | 114 |
| Severe adverse events | 7,852 | 6,282 | -4.67 | 114 |  | 9,422 | -4.67 | 114 |
| Mild adverse events | 199 | 159 | -4.67 | 114 |  | 239 | -4.67 | 114 |
| Low/Moderate intensity statin (annual) | 48 | 38 | -4.41 | 114 |  | 58 | -4.93 | 114 |
| High intensity statin (annual) | 91 | 73 | -5.17 | 114 |  | 109 | -4.17 | 114 |
| MI (post event, annual) | 508 | 406 | -4.66 | 114 |  | 609 | -4.68 | 114 |
| CABG or PCI (post event, annual) | 508 | 406 | -4.65 | 114 |  | 609 | -4.68 | 114 |
| Stroke (post event, annual) | 20,264 | 16,211 | -4.23 | 114 |  | 24,316 | -5.11 | 114 |
| Multiple CVD state (post event, annual) | 9,968 | 7,975 | -4.63 | 114 |  | 11,962 | -4.70 | 114 |
| Diabetes (annual) | 2,661 | 2,129 | -4.76 | 114 |  | 3,193 | -4.58 | 114 |
| **Annual event rates** |  |  |  |  |  |  |  |  |
| MI | 0.0037 | 0.0030 | -5.10 | 122 |  | 0.0044 | -4.32 | 109 |
| Stroke | 0.0034 | 0.0027 | -5.03 | 119 |  | 0.0041 | -4.37 | 110 |
| Revascularization | 0.0071 | 0.0057 | -3.95 | 104 |  | 0.0085 | -5.38 | 125 |
| Recurrent MI | 0.022 | 0.018 | -4.64 | 114 |  | 0.026 | -4.70 | 114 |
| MI post stroke | 0.0074 | 0.0059 | -4.66 | 114 |  | 0.0089 | -4.67 | 114 |
| Recurrent stroke | 0.023 | 0.018 | -4.67 | 114 |  | 0.028 | -4.67 | 114 |
| CVD post CABG | 0.0377 | 0.030 | -4.65 | 114 |  | 0.045 | -4.68 | 114 |
| CVD post PCI | 0.0615 | 0.049 | -4.57 | 114 |  | 0.074 | -4.77 | 115 |
| Death post MI | 0.0352 | 0.028 | -4.66 | 114 |  | 0.042 | -4.68 | 115 |
| Death post-stroke | 0.0649 | 0.052 | -4.69 | 113 |  | 0.078 | -4.65 | 116 |
| Death post CABG | 0.0239 | 0.019 | -4.67 | 114 |  | 0.029 | -4.67 | 115 |
| Death post PCI | 0.0295 | 0.024 | -4.68 | 113 |  | 0.035 | -4.66 | 116 |
| Death post multiple CVD | 0.1 | 0.08 | -4.67 | 114 |  | 0.12 | -4.67 | 115 |
| Death post severe adverse event | 0.09 | 0.072 | -4.67 | 114 |  | 0.108 | -4.67 | 114 |
| Diabetes from HST | 0.003 | 0.0024 | -4.82 | 125 |  | 0.0036 | -4.51 | 104 |
| Diabetes from MST | 0.001 | 0.0008 | -4.62 | 111 |  | 0.0012 | -4.71 | 118 |
| Mild adverse events from statin | 0.056 | 0.0001 | -5.18 | 120 |  | 0.175 | -3.59 | 102 |
| Severe adverse events from statin | 0.0001 | 0.00008 | -4.67 | 114 |  | 0.00012 | -4.67 | 114 |

In the base case analysis of a cohort of 100,000 intermediate-risk patients over a 5-year time horizon, the Test-and-HST strategy (Test and treat those in top decile of LDL-P with high-intensity statin therapy and treat everyone else with moderate-intensity statin therapy) cost $4.67MM less and had 115 more QALY than the MST strategy (treat all with moderate-intensity statin therapy).

**Table S3. Deterministic Sensitivity Analysis:** Incremental Cost and Incremental QALY per 100,000 patients for all parameters for the Test and MST strategy compared with the Do-Not-Treat strategy.

|  | |  | **Low range** | | |  | **High range** | | |
| --- | --- | --- | --- | --- | --- | --- | --- | --- | --- |
| **Parameter** | | **Base**  **Value** | **Value** | **Inc. Cost**  **($, MM)** | **Inc. Utility** |  | **Value** | **Inc. Cost**  **($, MM)** | **Inc. Utility** |
| **Effect of interventions** | |  |  |  |  |  |  |  |  |
| High-intensity statin (risk reduction) | |  |  |  |  |  |  |  |  |
| MI | 0.46 | 0.30 | -3.25 | 45 |  | 0.70 | -3.25 | 45 | |
| Revascularization | 0.54 | 0.41 | -3.25 | 45 |  | 0.72 | -3.25 | 45 | |
| Stroke | 0.52 | 0.34 | -3.25 | 45 |  | 0.79 | -3.25 | 45 | |
| Moderate-intensity statin (risk reduction) | |  |  |  |  |  |  |  |  |
| CHD | 0.75 | 0.71 | -4.43 | 61 |  | 0.78 | -2.36 | 32 | |
| Stroke | 0.83 | 0.76 | -3.99 | 56 |  | 0.87 | -2.82 | 38 | |
| **Ratios** | |  |  |  |  |  |  |  |  |
| Relative Risk LDL-P (per SD) | 1.4 | 1.12 | -.594 | 5 |  | 1.75 | -6.27 | 90 | |
| Fraction of CABG in revascularization | 0.2 | 0.16 | -3.25 | 45 |  | 0.24 | -3.24 | 44 | |
| Fraction of fatal MI among MI | 0.125 | 0.10 | -3.31 | 42 |  | 0.15 | -3.18 | 47 | |
| Fraction of fatal stroke among stroke | 0.132 | 0.11 | -3.29 | 43 |  | 0.16 | -3.20 | 46 | |
| Fraction discontinuing statin therapy | 0.31 | 0 | -6.01 | 67 |  | 0.44 | -1.18 | 28 | |
| **State utilities** | |  |  |  |  |  |  |  |  |
| Disease free taking statins | 0.998 | 0.991 | -3.25 | -181 |  | 1.0 | -3.25 | 109 | |
| Post-MI | 0.778 | 0.58 | -3.25 | 63 |  | 0.84 | -3.25 | 39 | |
| Post-Stroke | 0.768 | 0.46 | -3.25 | 61 |  | 0.82 | -3.25 | 42 | |
| Post-PCI or CABG | 0.768 | 0.52 | -3.25 | 92 |  | 0.83 | -3.25 | 33 | |
| Multiple CVD^†^ | 0.605 | 0.48 | -3.25 | 47 |  | 0.73 | -3.25 | 42 | |
| Diabetes | 0.8 | 0.71 | -3.25 | 37 |  | 0.84 | -3.25 | 48 | |
| Mild adverse events (disutility) | 0.005 | -0.006 | -3.25 | 44 |  | -0.004 | -3.25 | 45 | |
| Severe adverse events (disutility) | 0.038 | -0.046 | -3.25 | 44 |  | -0.030 | -3.25 | 45 | |
| **Costs (2014 USD)** | |  |  |  |  |  |  |  |  |
| LDL-P test | 42 | 34 | -4.09 | 45 |  | 51 | -2.40 | 45 | |
| Nonfatal MI (1^st^ year) | 69,819 | 55,855 | -2.54 | 45 |  | 83,783 | -3.95 | 45 | |
| Fatal MI | 19,373 | 15,499 | -3.21 | 45 |  | 23,248 | -3.28 | 45 | |
| Nonfatal stroke (1^st^ year) | 23,021 | 18,417 | -3.13 | 45 |  | 27,626 | -3.36 | 45 | |
| Fatal stroke | 11,951 | 9,561 | -3.23 | 45 |  | 14,341 | -3.26 | 45 | |
| CABG (1^st^ year) | 41,388 | 33,111 | -3.10 | 45 |  | 49,666 | -3.39 | 45 | |
| PCI (1^st^ year) | 38,998 | 31,198 | -2.71 | 45 |  | 46,798 | -3.79 | 45 | |
| Diabetes (diagnosis) | 138 | 111 | -3.25 | 45 |  | 166 | -3.25 | 45 | |
| Severe adverse events | 7,852 | 6,282 | -3.25 | 45 |  | 9,422 | -3.24 | 45 | |
| Mild adverse events | 199 | 159 | -3.27 | 45 |  | 239 | -3.22 | 45 | |
| Low/Moderate intensity statin (annual) | 48 | 38 | -3.51 | 45 |  | 58 | -2.99 | 45 | |
| High intensity statin (annual) | 91 | 73 | -3.25 | 45 |  | 109 | -3.25 | 45 | |
| MI (post event, annual) | 508 | 406 | -3.24 | 45 |  | 609 | -3.26 | 45 | |
| CABG or PCI (post event, annual) | 508 | 406 | -3.23 | 45 |  | 609 | -3.27 | 45 | |
| Stroke (post event, annual) | 20,264 | 16,211 | -3.00 | 45 |  | 24,316 | -3.50 | 45 | |
| Multiple CVD state (post event, annual) | 9,968 | 7,975 | -3.21 | 45 |  | 11,962 | -3.28 | 45 | |
| Diabetes (annual) | 2,661 | 2,129 | -3.29 | 45 |  | 3,193 | -3.20 | 45 | |
| **Annual event rates** | |  |  |  |  |  |  |  |  |
| MI | 0.0037 | 0.0030 | -3.63 | 51 |  | 0.0044 | -2.94 | 39 | |
| Stroke | 0.0034 | 0.0027 | -3.78 | 52 |  | 0.0041 | -2.80 | 38 | |
| Revascularization | 0.0071 | 0.0057 | -2.42 | 32 |  | 0.0085 | -4.07 | 57 | |
| Recurrent MI | 0.022 | 0.018 | -3.22 | 44 |  | 0.026 | -3.27 | 45 | |
| MI post stroke | 0.0074 | 0.0059 | -3.24 | 44 |  | 0.0089 | -3.25 | 45 | |
| Recurrent stroke | 0.023 | 0.018 | -3.25 | 44 |  | 0.028 | -3.25 | 45 | |
| CVD post CABG | 0.0377 | 0.030 | -3.23 | 44 |  | 0.045 | -3.27 | 45 | |
| CVD post PCI | 0.0615 | 0.049 | -3.12 | 44 |  | 0.074 | -3.37 | 45 | |
| Death post MI | 0.0352 | 0.028 | -3.24 | 44 |  | 0.042 | -3.26 | 45 | |
| Death post-stroke | 0.0649 | 0.052 | -3.26 | 44 |  | 0.078 | -3.24 | 45 | |
| Death post CABG | 0.0239 | 0.019 | -3.25 | 44 |  | 0.029 | -3.25 | 45 | |
| Death post PCI | 0.0295 | 0.024 | -3.26 | 43 |  | 0.035 | -3.24 | 46 | |
| Death post multiple CVD | 0.1 | 0.08 | -3.25 | 44 |  | 0.12 | -3.24 | 45 | |
| Death post severe adverse event | 0.09 | 0.072 | -3.25 | 45 |  | 0.108 | -3.25 | 44 | |
| Diabetes from HST | 0.003 | 0.0024 | -3.25 | 45 |  | 0.0036 | -3.25 | 45 | |
| Diabetes from MST | 0.001 | 0.0008 | -3.29 | 48 |  | 0.0012 | -3.20 | 41 | |
| Mild adverse events from statin | 0.056 | 0.0001 | -3.78 | 48 |  | 0.175 | -2.10 | 36 | |
| Severe adverse events from statin | 0.0001 | 0.00008 | -3.25 | 45 |  | 0.00012 | -3.24 | 44 | |

In the base case analysis of a cohort of 100,000 intermediate-risk patients over a 5-year time horizon, the Test-and-MST strategy (test and treat those in top decile of LDL-P with moderate-intensity statin therapy) cost $3.25 MM less and had 44 more QALY than the Do-Not-Treat (do not treat anyone with statin therapy) strategy.

**Table S4. Deterministic Sensitivity Analysis:** Incremental Cost and Incremental QALY per 100,000 patients for all parameters for the HST strategy compared with the MST strategy.

|  |  | **Low range** | | |  | **High range** | | |
| --- | --- | --- | --- | --- | --- | --- | --- | --- |
| **Parameter** | **Base**  **Value** | **Value** | **Inc. Cost**  **($, MM)** | **Inc. Utility** |  | **Value** | **Inc. Cost**  **($, MM)** | **Inc. Utility** |
| **Effect of interventions** |  |  |  |  |  |  |  |  |
| High-intensity statin (risk reduction) |  |  |  |  |  |  |  |  |
| MI | 0.46 | 0.30 | -56.53 | 659 |  | 0.70 | -27.15 | 291 |
| Revascularization | 0.54 | 0.41 | -57.78 | 700 |  | 0.72 | -26.79 | 250 |
| Stroke | 0.52 | 0.34 | -55.99 | 680 |  | 0.79 | -27.94 | 259 |
| Moderate-intensity statin (risk reduction) |  |  |  |  |  |  |  |  |
| CHD | 0.75 | 0.71 | -37.88 | 417 |  | 0.78 | -49.91 | 582 |
| Stroke | 0.83 | 0.76 | -40.42 | 446 |  | 0.87 | -47.23 | 549 |
| **Ratios** |  |  |  |  |  |  |  |  |
| Relative Risk LDL-P (per SD) | 1.4 | 1.12 | -44.63 | 509 |  | 1.75 | -45.03 | 517 |
| Fraction of CABG in revascularization | 0.2 | 0.16 | -44.77 | 512 |  | 0.24 | -44.74 | 511 |
| Fraction of fatal MI among MI | 0.125 | 0.10 | -45.19 | 497 |  | 0.15 | -44.32 | 525 |
| Fraction of fatal stroke among stroke | 0.132 | 0.11 | -45.23 | 497 |  | 0.16 | -44.28 | 525 |
| Fraction discontinuing statin therapy | 0.31 | 0 | -61.63 | 750 |  | 0.44 | -32.14 | 333 |
| **State utilities** |  |  |  |  |  |  |  |  |
| Disease free taking statins | 0.998 | 0.991 | -44.76 | 506 |  | 1.0 | -44.76 | 513 |
| Post-MI | 0.778 | 0.58 | -44.76 | 638 |  | 0.84 | -44.76 | 471 |
| Post-Stroke | 0.768 | 0.46 | -44.76 | 688 |  | 0.82 | -44.76 | 484 |
| Post-PCI or CABG | 0.768 | 0.52 | -44.76 | 743 |  | 0.83 | -44.76 | 457 |
| Multiple CVD^†^ | 0.605 | 0.48 | -44.76 | 523 |  | 0.73 | -44.76 | 499 |
| Diabetes | 0.8 | 0.71 | -44.76 | 349 |  | 0.84 | -44.76 | 589 |
| Mild adverse events (disutility) | 0.005 | -0.006 | -44.76 | 511 |  | -0.004 | -44.76 | 511 |
| Severe adverse events (disutility) | 0.038 | -0.046 | -44.76 | 511 |  | -0.030 | -44.76 | 511 |
| **Costs (2014 USD)** |  |  |  |  |  |  |  |  |
| LDL-P test | 42 | 34 | -44.76 | 511 |  | 51 | -44.76 | 511 |
| Nonfatal MI (1^st^ year) | 69,819 | 55,855 | -40.17 | 511 |  | 83,783 | -49.34 | 511 |
| Fatal MI | 19,373 | 15,499 | -44.54 | 511 |  | 23,248 | -44.97 | 511 |
| Nonfatal stroke (1^st^ year) | 23,021 | 18,417 | -43.49 | 511 |  | 27,626 | -46.02 | 511 |
| Fatal stroke | 11,951 | 9,561 | -44.59 | 511 |  | 14,341 | -44.93 | 511 |
| CABG (1^st^ year) | 41,388 | 33,111 | -44.05 | 511 |  | 49,666 | -45.46 | 511 |
| PCI (1^st^ year) | 38,998 | 31,198 | -42.09 | 511 |  | 46,798 | -47.42 | 511 |
| Diabetes (diagnosis) | 138 | 111 | -44.77 | 511 |  | 166 | -44.74 | 511 |
| Severe adverse events | 7,852 | 6,282 | -44.76 | 511 |  | 9,422 | -44.75 | 511 |
| Mild adverse events | 199 | 159 | -44.76 | 511 |  | 239 | -44.76 | 511 |
| Low/Moderate intensity statin (annual) | 48 | 38 | -42.09 | 511 |  | 58 | -47.42 | 511 |
| High intensity statin (annual) | 91 | 73 | -49.82 | 511 |  | 109 | -39.69 | 511 |
| MI (post event, annual) | 508 | 406 | -44.69 | 511 |  | 609 | -44.82 | 511 |
| CABG or PCI (post event, annual) | 508 | 406 | -44.66 | 511 |  | 609 | -44.85 | 511 |
| Stroke (post event, annual) | 20,264 | 16,211 | -42.22 | 511 |  | 24,316 | -47.29 | 511 |
| Multiple CVD state (post event, annual) | 9,968 | 7,975 | -44.56 | 511 |  | 11,962 | -44.95 | 511 |
| Diabetes (annual) | 2,661 | 2,129 | -45.69 | 511 |  | 3,193 | -43.82 | 511 |
| **Annual event rates** |  |  |  |  |  |  |  |  |
| MI | 0.0037 | 0.0030 | -47.19 | 551 |  | 0.0044 | -42.78 | 479 |
| Stroke | 0.0034 | 0.0027 | -46.80 | 538 |  | 0.0041 | -43.06 | 489 |
| Revascularization | 0.0071 | 0.0057 | -40.71 | 451 |  | 0.0085 | -48.78 | 571 |
| Recurrent MI | 0.022 | 0.018 | -44.57 | 511 |  | 0.026 | -44.94 | 512 |
| MI post stroke | 0.0074 | 0.0059 | -44.72 | 511 |  | 0.0089 | -44.79 | 512 |
| Recurrent stroke | 0.023 | 0.018 | -44.74 | 511 |  | 0.028 | -44.77 | 512 |
| CVD post CABG | 0.0377 | 0.030 | -44.66 | 511 |  | 0.045 | -44.85 | 512 |
| CVD post PCI | 0.0615 | 0.049 | -44.16 | 509 |  | 0.074 | -45.33 | 513 |
| Death post MI | 0.0352 | 0.028 | -44.70 | 507 |  | 0.042 | -44.81 | 515 |
| Death post-stroke | 0.0649 | 0.052 | -44.88 | 504 |  | 0.078 | -44.64 | 518 |
| Death post CABG | 0.0239 | 0.019 | -44.76 | 510 |  | 0.029 | -44.75 | 513 |
| Death post PCI | 0.0295 | 0.024 | -44.80 | 504 |  | 0.035 | -44.71 | 519 |
| Death post multiple CVD | 0.1 | 0.08 | -44.77 | 511 |  | 0.12 | -44.74 | 512 |
| Death post severe adverse event | 0.09 | 0.072 | -44.76 | 511 |  | 0.108 | -44.76 | 511 |
| Diabetes from HST | 0.003 | 0.0024 | -46.25 | 617 |  | 0.0036 | -43.26 | 406 |
| Diabetes from MST | 0.001 | 0.0008 | -44.29 | 477 |  | 0.0012 | -45.22 | 546 |
| Mild adverse events from statin | 0.056 | 0.0001 | -47.21 | 537 |  | 0.175 | -39.54 | 456 |
| Severe adverse events from statin | 0.0001 | 0.00008 | -44.76 | 511 |  | 0.00012 | -44.75 | 511 |

In the base case analysis of a cohort of 100,000 intermediate-risk patients over a 5-year time horizon, the HST (treat all with high-intensity statin therapy) strategy cost $44.76 MM less and had 512 more QALY than the MST (treat all with moderate-intensity statin therapy) strategy.
